# Supplementary figures and images for: The genetic paradigms of dietary restriction fail to extend life span in cep-1(gk138) mutant of C. elegans p53 due to possible background mutations
Source: PLoS One. 2020 Nov 12;15(11):e0241478. doi: 10.1371/journal.pone.0241478 (PMC7660490; doi:10.1371/journal.pone.0241478)

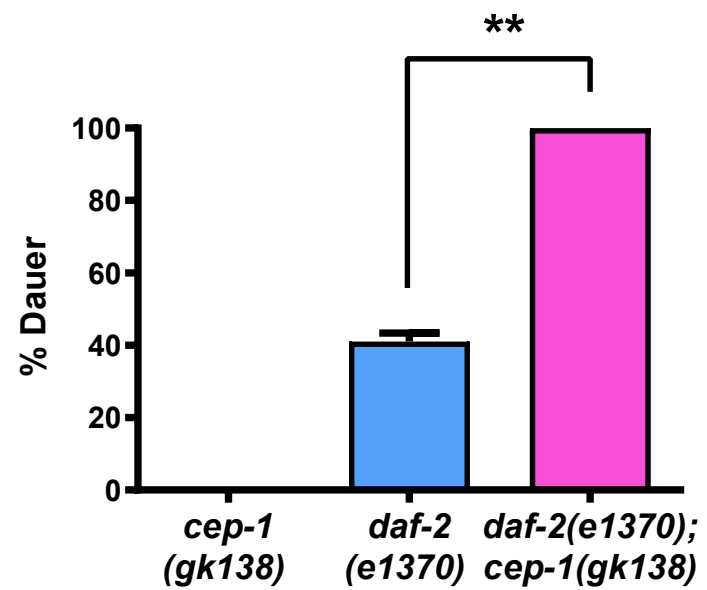

S1 Fig.

Supplement: S1 Fig — Dauer assay was performed at 22.5 °C. Data are presented as mean values ± SEM. N = 2 independent experiments. Unpaired two-tailed t-test was used for statistical analysis. **P≤0.01. (PDF) [file pone.0241478.s001.pdf]

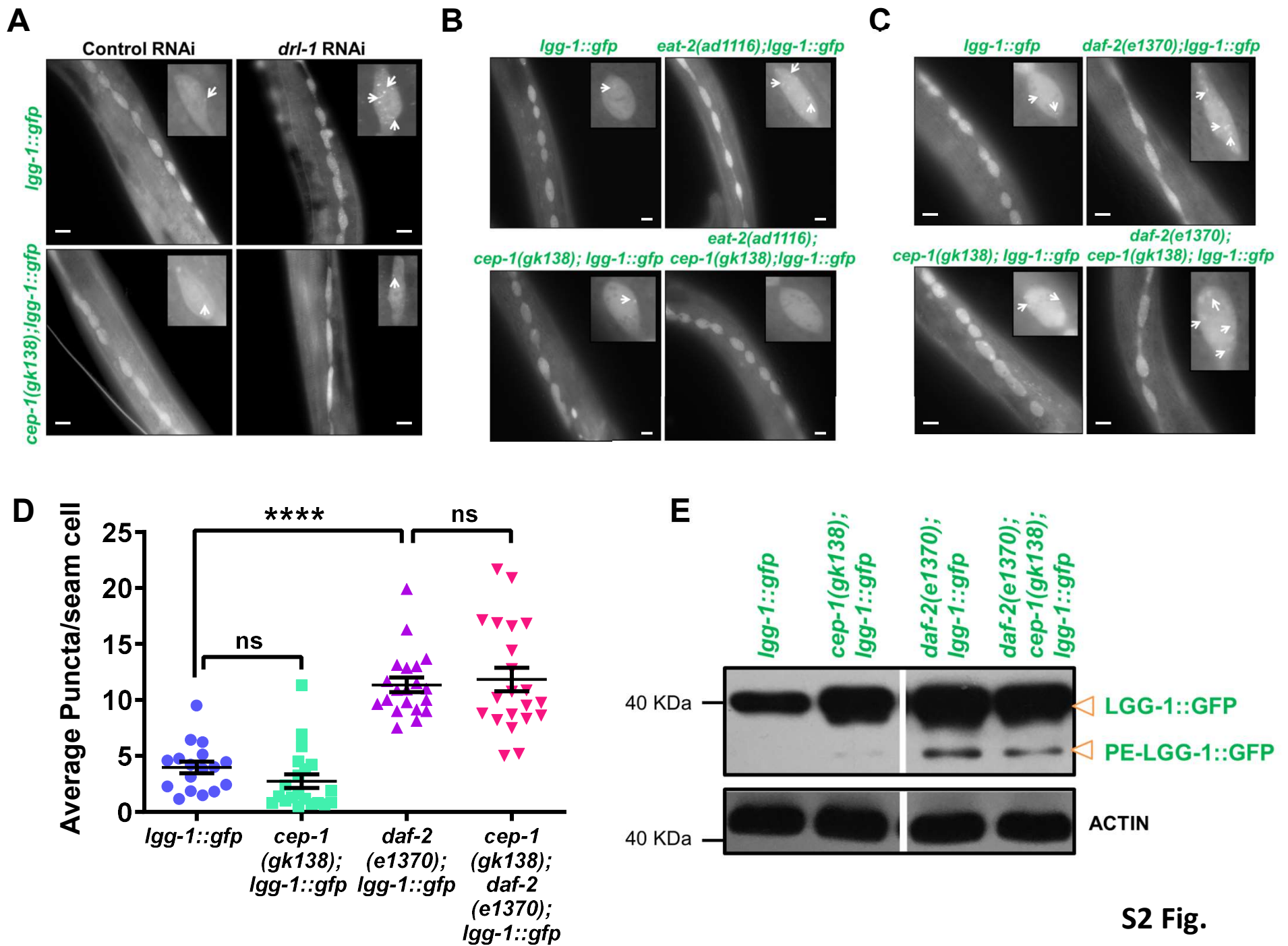

S2 Fig.

Supplement: S2 Fig — Representative images showing autophagosomes as GFP puncta in the hypodermal seam cells of L3-staged (A) lgg::gfp and cep-1(gk138);lgg::gfp worms on control or drl-1 RNAi, (B) eat-2(ad116);lgg-1::gfp and eat-2(ad116);cep-1(gk138);lgg-1:gfp, (C) daf-2(e1370);lgg-1::gfp and daf-2(e1370);cep-1(gk138);lgg-1::gfp. Images were captured at 630X magnification. Inset images represent zoomed-in areas showing one seam cell. Arrows point to autophagosome puncta. Scale bar is 10 μm. (D) The increased autophagosome formation in daf-2(e1370);lgg-1:gfp is unaffected in daf-2(e1370);cep-1(gk138);lgg-1::gfp. Quantification of GFP puncta for one of two independent experiments is shown. Data are presented as mean values ± SEM. N = 2 independent experiments. No. of animals analysed n≥17. Unpaired two-tailed t-test with Welch’s correction was used for statistical analysis. ****P≤0.0001, ns = non-significant. Source data is provided as source data file. (E) Western blot using anti-GFP antibody to detect the unmodified LGG-1 or PE-LGG-1 in daf-2(e1370);lgg-1::gfp and daf-2(e1370);cep-1(gk138);lgg-1::gfp. β-ACTIN was used as a loading control. One representative blot out of four independent experiments is shown. Experiments were performed at 20°C. Uncropped blots are provided in Supplementary Information file. (PDF) [file pone.0241478.s002.pdf]

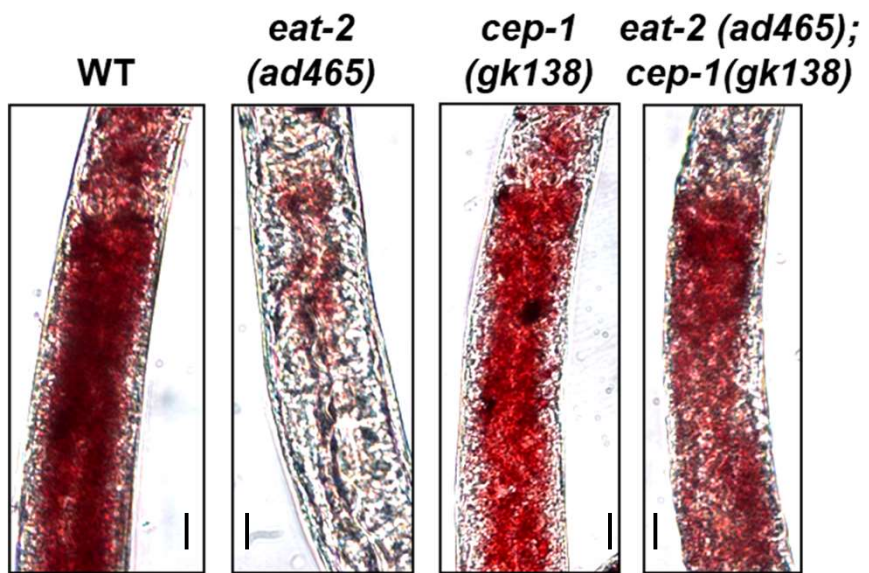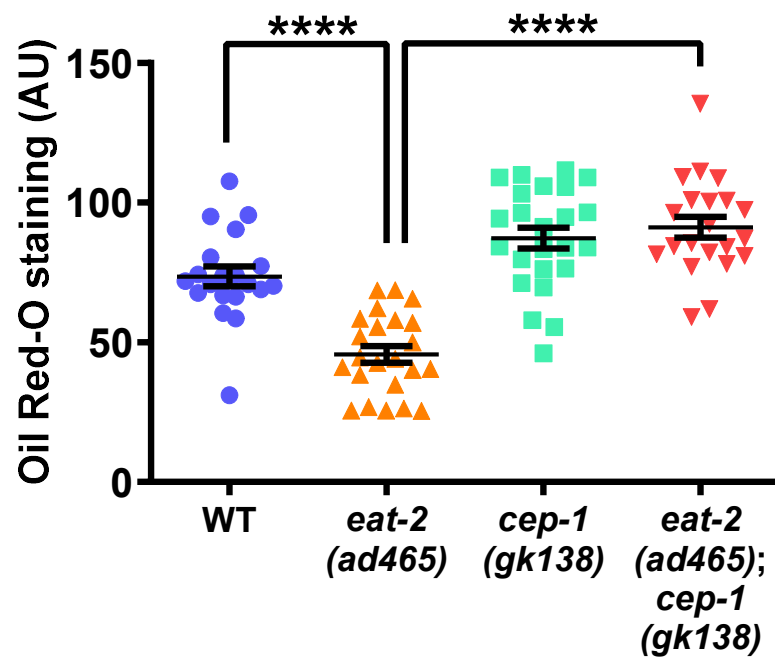

S3 Fig.

Supplement: S3 Fig — Lowering of fat storage in eat-2(ad465) is attenuated in eat-2(ad465);cep-1(gk138). Representative images (upper panel) and quantification (lower panel) for one of four independent experiments is shown. Images were captured at 400X magnification. Scale bar is 20 μm. Data are presented as mean values ± SEM. N = 2 independent experiments. No. of animals analysed n≥20. Unpaired two-tailed t-test with Welch’s correction was used for statistical analysis. ****P≤0.0001. Experiments were performed at 20 °C. Source data is provided as source data file. (PDF) [file pone.0241478.s003.pdf]

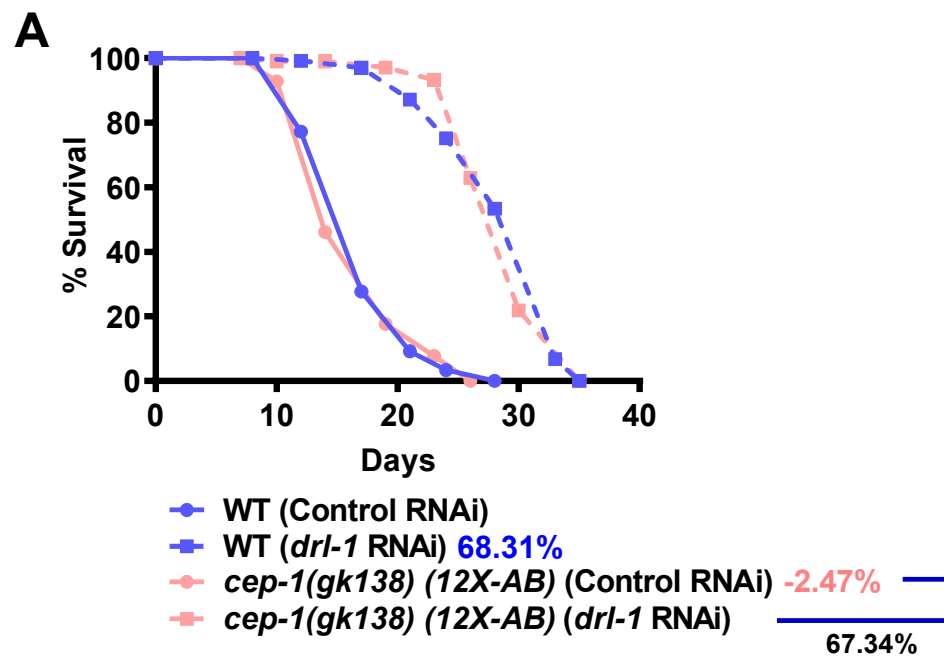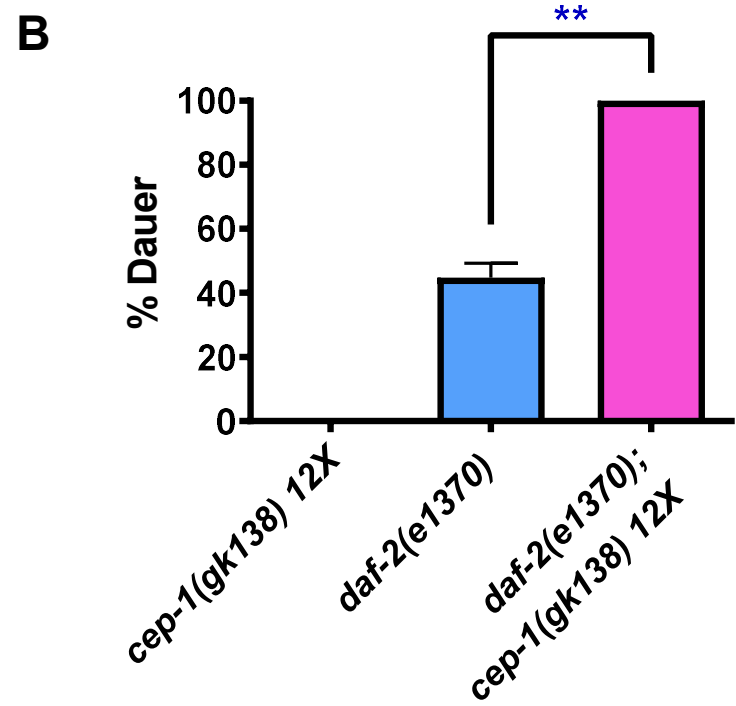

S4 Fig.

Supplement: S4 Fig — (A) Life span analysis of cep-1(gk138) (12X-AB) generated earlier [20] as well as WT, grown on control or drl-1 RNAi. Life span was performed at 20°C and details are provided in S1 Table. Mantel-Cox log rank test using OASIS software available at http://sbi.postech.ac.kr/oasis [22] was used for statistical analysis. (B) The percentage of dauer formed in daf-2(e1370) is enhanced in the genetic double daf-2(e1370);cep-1(gk138) 12X. Dauer assay was performed at 22.5 °C. Data are presented as mean values ± SEM. N = 3 independent experiments. Unpaired two-tailed t-test was used for statistical analysis. **P≤0.01. (PDF) [file pone.0241478.s004.pdf]
