# Supplementary material for: The genetic paradigms of dietary restriction fail to extend life span in cep-1(gk138) mutant of C. elegans p53 due to possible background mutations
Source: PLoS One. 2020 Nov 12;15(11):e0241478. doi: 10.1371/journal.pone.0241478 (PMC7660490; doi:10.1371/journal.pone.0241478)
Supplement: S2 Table — (DOCX) [file pone.0241478.s006.docx]

S2 Table: List of qRT-PCR primers used

| **Gene Name (Target)** | **Primer name** | **Sequence** |
| --- | --- | --- |
|  |  |  |
| *lgg-1* | Forward | GGT CCC ATC CGA TCT TAC TGT |
| *lgg-1* | Reverse | CGT GAT GGT CCT GGT AGA GT |
|  |  |  |
| *bec-1* | Forward | GAA AGA GCT CAA GGA TCG AAA CA |
| *bec-1* | Reverse | GAG CAT TAG AGC CAT TGC ACG |
|  |  |  |
| *unc-51* | Forward | CGC TAT GTT GAT CGC ACA GAC |
| *unc-51* | Reverse | GTG CAT TTG AGT AGG CCC AC |
|  |  |  |
| *vps-34* | Forward | CCT TAT TTG GTT CTC TCC ACA G |
| *vps-34* | Reverse | CCA TCT CCT GGA CGA AGT TC |
|  |  |  |
| *ugt-16* | Forward | CTTGCTGACGATCGACTAACC |
| *ugt-16* | Reverse | CGGTCTGTATGGCTTCTCTAAG |
|  |  |  |
| *gst-6* | Forward | CAAAAATAACACTCCATTC |
| *gst-6* | Reverse | GCCGCCTCGGTGTCATTTTGTC |
|  |  |  |
| *gst-28* | Forward | GCTTAAAGACGGCGCCCC |
| *gst-28* | Reverse | CCAGCATATCCGAATTTGTTGGC |
|  |  |  |
| *cyp-32B1* | Forward | GGTGTGTTGAAGTTATGGTTGGGACC |
| *cyp-32B1* | Reverse | TGTCGCCGGTGCTGATTAAAAGAC |
|  |  |  |
| *cyp-33C8* | Forward | CGCTGGATGATGTGCTCAACTACTGG |
| *cyp-33C8* | Reverse | GCTTCTTCTGCTCTTTCAGGTAGG |
|  |  |  |
| *cyp-34A4* | Forward | GATTTGAACAGGGTGACCCAGAAT |
| *cyp-34A4* | Reverse | TCGATGACATGCTCACCACT |
|  |  |  |
| *cyp-37B1* | Forward | GCTTGGAACGGGACTATTGAC |
| *cyp-37B1* | Reverse | TTGTTCGAGGAAAACCTTGGCCTG |
|  |  |  |
| *actin* | Forward | CTCTTGCCCCATCAACCATG |
| *actin* | Reverse | CTTGCTTGGAGATCCACATC |
