# Supplementary material for: The genetic paradigms of dietary restriction fail to extend life span in cep-1(gk138) mutant of C. elegans p53 due to possible background mutations
Source: PLoS One. 2020 Nov 12;15(11):e0241478. doi: 10.1371/journal.pone.0241478 (PMC7660490; doi:10.1371/journal.pone.0241478)
Supplement: S1 Raw images — (PDF) [file pone.0241478.s008.pdf]

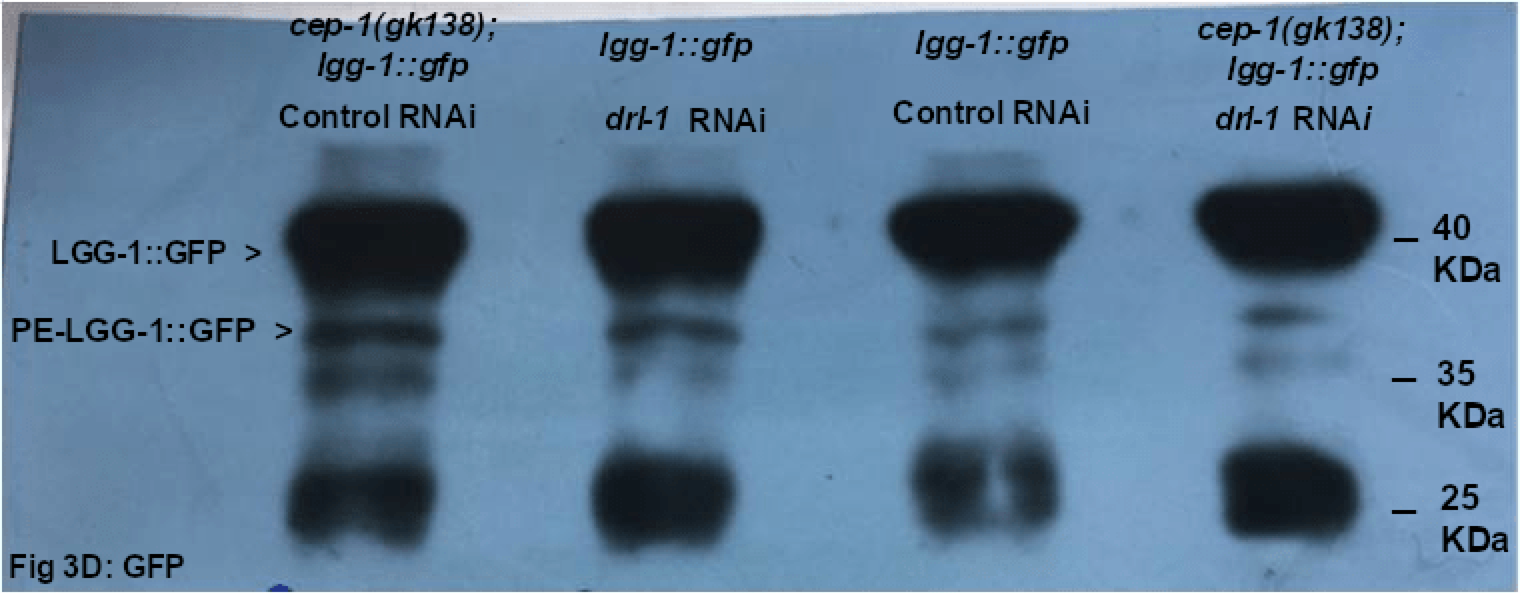

cep-1(gk138); lgg-1::gfp
Control RNAi
lgg-1::gfp
drl-1 RNAi
lgg-1::gfp
Control RNAi
cep-1(gk138); lgg-1::gfp
drl-1 RNAi
LGG-1::GFP >
PE-LGG-1::GFP >
40 KDa
35 KDa
25 KDa

Fig 3D: GFP

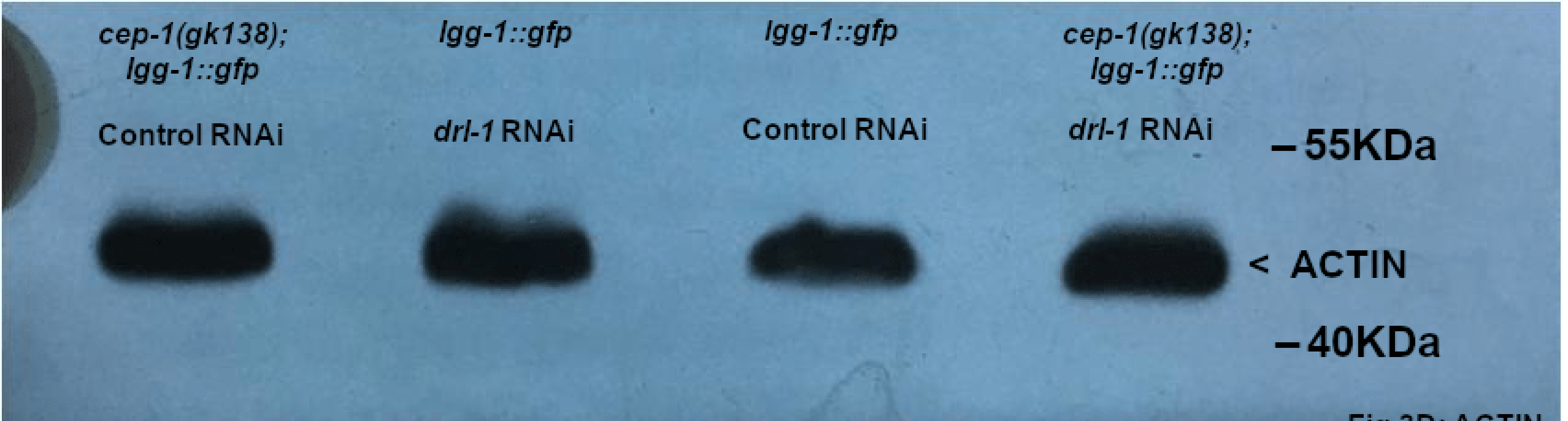

cep-1(gk138); lgg-1::gfp
Control RNAi
lgg-1::gfp
drl-1 RNAi
lgg-1::gfp
Control RNAi
cep-1(gk138); lgg-1::gfp
drl-1 RNAi
– 55KDa
< ACTIN
– 40KDa

Fig 3D: ACTIN

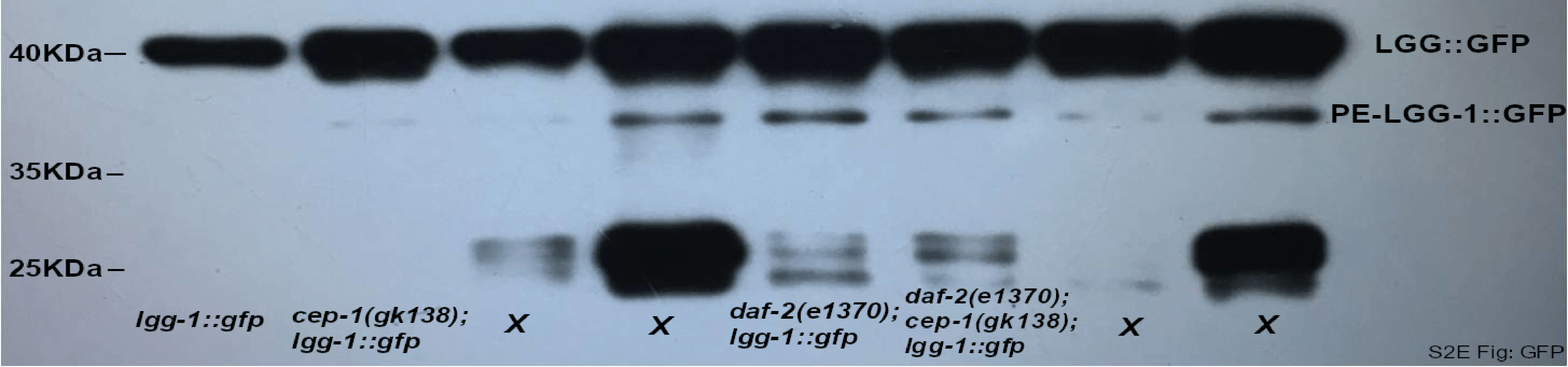
40KDa
35KDa
25KDa
LGG::GFP
PE-LGG-1::GFP
lgg-1::gfp
cep-1(gk138);
lgg-1::gfp
X
X
daf-2(e1370);
lgg-1::gfp
daf-2(e1370);
cep-1(gk138);
lgg-1::gfp
X
X
S2E Fig: GFP

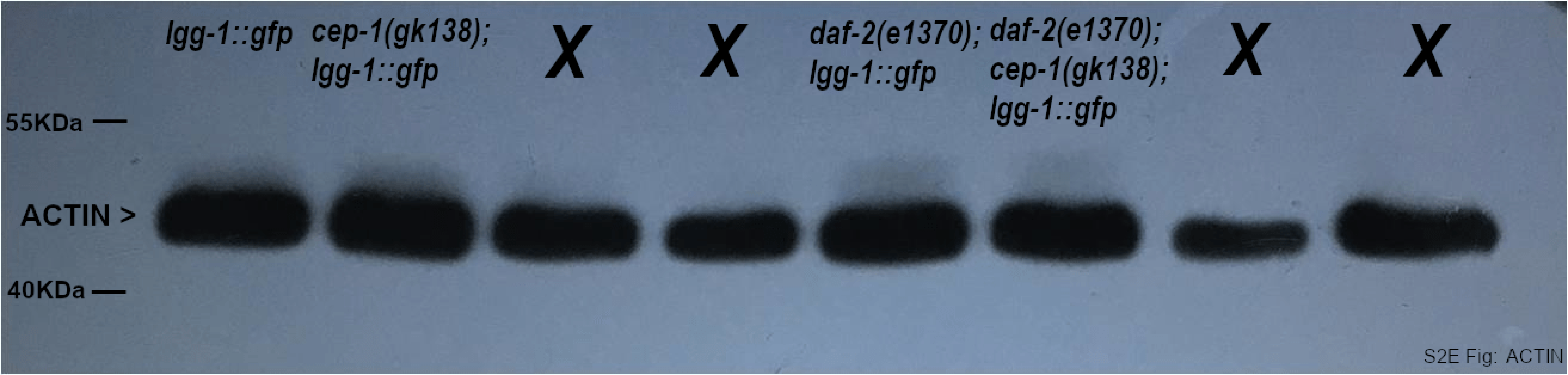

S2E Fig: ACTIN
