# Supplementary material for: The genetic paradigms of dietary restriction fail to extend life span in cep-1(gk138) mutant of C. elegans p53 due to possible background mutations
Source: PLoS One. 2020 Nov 12;15(11):e0241478. doi: 10.1371/journal.pone.0241478 (PMC7660490; doi:10.1371/journal.pone.0241478)
Supplement: Attachment — Submitted filename: Comments.pdf [file pone.0241478.s009.pdf]

The genetic paradigms of dietary restriction fail to extend life span in cep-1(gk138) mutant of C. elegans p53 due to possible background mutations

Anita Goyala, Aiswarya Baruah and Arnab Mukhopadhyay

Summary: In the current manuscript, Goyala et al. investigate the role of a *C. elegans* ortholog of p53, *cep-1*, in DR-mediated longevity assurance. The authors show TJ1 *cep-1(gk138)* strain prevents genetic modes of DR from increasing life span, the effect may be the result of background mutation(s). This study is well designed, and the findings are clear. However, there are still some concerns that need to be clarified.

Minor

1. There are several typos and grammatical mistakes throughout the manuscript. The authors should check the manuscript carefully. A few examples are listed below:
   - Line 25, Please remove the word "etc" and mention the names. Also correct/rewrite the sentence "Including an increase in expression of cytoprotective genes, better proteostasis, etc."
   - Please rewrite the sentence "We find that in cep-1(gk138), two aspects of DR, increased autophagy and the up-regulation of expression of cytoprotective xenobiotic detoxification program (cXDP) genes are dampened"
   - Line 44, "Research over the past decades, many using *Caenorhabditis elegans"*, Please remove many.
   - Line 60, "For e.g.," Remove e.g. and replace it with example.
2. Please add statistical information in Figure legend 1 and 6.
3. Figure 3, quantification of GFP puncta averaged over 2 biological repeats. The authors should perform at least 3 biological repeats.
4. Figure S2 A-C, and 5C, please add a scale bar and mention the protein band size for figure S2E.
5. Authors have used "Fig" and "Figure" both. Please make it uniform throughout the manuscript. For example, Fig 4 and figure 5.
6. Figure 4, quantification of Oil Red O staining averaged over two biological replicates, please add at least one more replicate.
7. Please separate the main figures/legends and supplementary. For example, first add main figures 1-6, followed by supplementary figures 1-4.
8. Please add details for all Experimental procedures such as for C.elegans culture.
9. The molecular weight marker in all blots is missing. The authors should provide full-length western blots in supplementary and mention the protein band size.
