# Supplementary material for: The genetic paradigms of dietary restriction fail to extend life span in cep-1(gk138) mutant of C. elegans p53 due to possible background mutations
Source: PLoS One. 2020 Nov 12;15(11):e0241478. doi: 10.1371/journal.pone.0241478 (PMC7660490; doi:10.1371/journal.pone.0241478)
Supplement: Attachment — Submitted filename: Goyala.pdf [file pone.0241478.s010.pdf]

The manuscript by Goyala et al examines the role of CEP-1, ortholog of mammalian p53, in the regulation of dietary restriction (DR) mediated increase in life span of *C. elegans*. The authors show that CEP-1 differentially regulates longevity induced by DR through genetic means versus diet or bacterial dilution. This has been shown before and mechanisms behind this have been elucidated in some detail already. Some of these have been referenced in the introduction part of the manuscript. Many papers have been published linking CEP-1 to autophagy and to mutations in electron transport chain. Authors use well established assays to study autophagy, neutral lipid levels and longevity in context of *cep-1* mutation in this study. The authors contend that a background mutation in *cep-1* mutant is responsible for phenotypes linked to autophagy and DR mediated longevity. I agree with authors that a background mutation might well be responsible for the difference in phenotype observed in 10X backcrossed versus 12X backcrossed *cep-1* mutant. Unfortunately, the authors make no attempt to identify the background mutation say by genomics approaches followed by validation using RNAi nor do they attempt to segregate the mutation from *cep-1* mutation by crosses and QTL analyses. Without identifying the mutation (one or more) and their direct effect on mechanism of longevity, this study does not add any value to the community interested in longevity and DR specifically or *C. elegans* biology in general. Therefore, this study does not merit publication.

P53 is considered the guardian of the genome and it is not difficult to envisage that mutations may arise at a higher frequency in *cep-1* mutant than in wild type strain. Going forward, this gives the authors an excellent opportunity to study mutation(s) due to the lack of CEP-1 activity.

Other Comments

1. Life span assays: None of the life span experiments have been performed more than 2 times. Authors must perform these experiments at least three and preferably more number of times.
2. Figure 4: Discrepancy between ORO stained worms and their quantification. drl-1 RNAi and eat-2 mutation appears to cause almost complete loss of ORO stained lipids but the quantification indicates that 50-60% of lipid are present. The authors must check their method for quantification and quantify lipids biochemically.
